# Xanthine oxidoreductase inhibition ameliorates high glucose-induced glomerular endothelial injury by activating AMPK through the purine salvage pathway

**DOI:** 10.1038/s41598-024-61436-1

**Published:** 2024-05-15

**Authors:** Keum-Jin Yang, Hwajin Park, Yoon-Kyung Chang, Cheol Whee Park, Suk Young Kim, Yu Ah Hong

**Affiliations:** 1 Clinical Research Institute, Daejeon St. Mary’s Hospital, Daejeon, Republic of Korea; 2 Division of Nephrology, Department of Internal Medicine, College of Medicine, The Catholic University of Korea, Seoul, Republic of Korea

**Keywords:** High glucose, Oxidative stress, Xanthine oxidoreductase, AMPK, Purine salvage pathway, Molecular medicine, Nephrology, Kidney diseases

## Abstract

Xanthine oxidoreductase (XOR) contributes to reactive oxygen species production. We investigated the cytoprotective mechanisms of XOR inhibition against high glucose (HG)-induced glomerular endothelial injury, which involves activation of the AMP-activated protein kinase (AMPK). Human glomerular endothelial cells (GECs) exposed to HG were subjected to febuxostat treatment for 48 h and the expressions of AMPK and its associated signaling pathways were evaluated. HG-treated GECs were increased xanthine oxidase/xanthine dehydrogenase levels and decreased intracellular AMP/ATP ratio, and these effects were reversed by febuxostat treatment. Febuxostat enhanced the phosphorylation of AMPK, the activation of peroxisome proliferator-activated receptor (PPAR)-gamma coactivator (PGC)-1α and PPAR-α and suppressed the phosphorylation of forkhead box O (FoxO)3a in HG-treated GECs. Febuxostat also decreased nicotinamide adenine dinucleotide phosphate oxidase (Nox)1, Nox2, and Nox4 expressions; enhanced superoxide dismutase activity; and decreased malondialdehyde levels in HG-treated GECs. The knockdown of AMPK inhibited PGC-1α–FoxO3a signaling and negated the antioxidant effects of febuxostat in HG-treated GECs. Despite febuxostat administration, the knockdown of hypoxanthine phosphoribosyl transferase 1 (HPRT1) also inhibited AMPK–PGC-1α–FoxO3a in HG-treated GECs. XOR inhibition alleviates oxidative stress by activating AMPK–PGC-1α–FoxO3a signaling through the HPRT1-dependent purine salvage pathway in GECs exposed to HG conditions.

## Introduction

Diabetic kidney disease (DKD) is a major long-term severe microvascular complication of diabetes mellitus and the current leading cause of end-stage kidney disease^1^. Sustained exposure to hyperglycemia stimulates excessive production of reactive oxygen species (ROS) in the kidneys, which can lead to the development and progression of DKD. In addition to hyperglycemia, multifactorial pathophysiological abnormalities, such as hemodynamic mediators, metabolic disturbances, inflammation, and fibrosis, are involved in the pathogenesis of DKD^2^. Although the underlying molecular mechanisms of DKD are complex and not fully elucidated, an imbalance between the generation of ROS and antioxidant defense mechanisms plays a pivotal role in the development and progression of DKD^3^. Oxidative stress derived from excessive ROS via several endogenous pathways and potential interplay between ROS sources could lead to endothelial dysfunction caused by glomerular endothelial cell (GEC) injury, which has been suggested to contribute to the early pathogenesis of DKD^4^.

Uric acid is the ultimate oxidation product resulting from the metabolic breakdown of purine nucleotides^5^. Xanthine oxidoreductase (XOR), comprising xanthine dehydrogenase (XDH) and xanthine oxidase (XO), regulates the pivotal rate-limiting process in the degradation of adenosine triphosphate (ATP) and purines. This enzymatic activity involves the oxidative hydroxylation of hypoxanthine/xanthine to xanthine/uric acid, leading to the generation of ROS^6^. High glucose environment could induce XOR-derived ROS in GECs, and lead to the disruption of glomerular endothelial permeability and homeostasis in the kidney^7,8^. Previous study suggested that XOR-derived ROS may contribute to the pathogenesis of DKD by intensifying oxidative stress and the inflammatory response via activation of the nuclear factor-κB (NF-κB) pathway^9^. Clinical studies have also proposed a significant association between elevated XOR activity and poor glycemic control^10,11^. Moreover, high XOR activity is independently correlated with vascular endothelial dysfunction in patients with diabetes mellitus^12^. Therefore, XOR inhibition may play a more promising role in preventing DKD by reducing ROS production and oxidative stress rather than simply controlling uric acid levels.

Adenosine monophosphate (AMP)-activated protein kinase (AMPK) is a key regulator of cellular metabolism involved in the activation of energy-producing pathways while inhibiting energy-consuming pathways in response to cellular stress^13,14^. Our previous study demonstrated that XOR inhibition hinders contrast-induced acute kidney injury (AKI) by ameliorating oxidative stress through the activation of AMPK and its downstream signaling pathway^15^. However, we did not reveal the precise mechanism of AMPK activation by XOR inhibition in that previous experiment. This study aimed to elucidate the cytoprotective effects of XOR inhibition on GEC injury and the precise mechanism of AMPK activation associated with the purine salvage pathway by XOR inhibition using an in vitro model of DKD involving high glucose (HG)-treated human GECs. In addition, another aim of this study is to investigate whether febuxostat, a potent and selective XOR inhibitor, has a beneficial role in mitigating GEC injury through this novel mechanism and could suggest its potential as a therapeutic candidate for preventing GEC injury in DKD.

## Methods

### Study design for in vitro experiments

Primary human GECs (Cell Systems, Kirkland, WA, USA) were grown and passaged in Angio–Proteome medium supplemented with 10% fetal bovine serum, 50 U/mL penicillin, and 50 μg/mL streptomycin. GECs were cultured under normal glucose conditions (low glucose: LG, 5.6 mmol/L glucose + 27.5 mmol/L mannitol) or HG conditions (33 mmol/L glucose) for 48 h. Febuxostat was obtained from SK Chemical (Sungnam, Gyeonggi-do, Republic of Korea) and dissolved in dimethyl sulfoxide. The GECs were divided into four groups: the LG group (LG), the LG group treated with febuxostat (LG + Feb), the HG group, and the HG group treated with febuxostat (HG + Feb). The treatment dose of febuxostat was determined to be 0.25 μM, a dose that increases cell viability in HG-treated GECs and activates AMPK based on the results of previous study^15^.

Silencer siRNAs targeting AMPKα1 or AMPKα2 (Thermo Fisher Scientific, Pleasanton, CA, USA) or a siRNA control (Bioneer, Daejeon, Republic of Korea) were transfected using Lipofectamine RNAiMax reagent (Thermo Fisher Scientific) following the manufacturer’s instructions to evaluate the direct effect of AMPK activation on XOR inhibition. The GECs were transfected with 10 nM AMPKα1 and 5 nM AMPKα2 siRNAs with transfection reagent for 24 h in Opti-MEM (Thermo Fisher Scientific). After transfection, GECs were treated with febuxostat in HG media to investigate the direct effects of AMPK downregulation.

To assess the direct effect of hypoxanthine phosphoribosyltransferase 1 (HPRT1) inhibition on AMPK activation, 10 nM silencer siRNAs targeting HPRT1 (Thermo Fisher Scientific) or a control siRNA (Bioneer) was transfected for 24 h as described above. After transfection, GECs were treated with febuxostat in HG medium to investigate the direct effects of HPRT1 inhibition.

### MTT assay

Cell viability was assessed after 48 h of exposure to LG or HG media supplemented with febuxostat by the MTT (3-[4,5-dimethyl(thiazol-2-yl)-3,5-dipheryl] tetradium bromide) assay (EZ-Cytox; Daeil Lab Service, Cheongwon, Chungcheongbuk-do, Republic of Korea). The optical density (OD) of the samples was determined at 450 nm with a microplate reader (Bio-Rad Laboratories, Hercules, CA, USA).

### Immunoblot analyses

To analyze the changes in the expression of AMPK and its downstream signaling pathway by febuxostat treatment in HG-treated GECs, immunoblot analysis was performed using the following primary antibodies: total AMPK, phospho-Thr^172^ AMPK (Cell Signaling Technology, Danvers, MA, USA), peroxisome proliferator–activated receptor (PPAR)-gamma coactivator (PGC)-1α (Boster Biological Technology, Pleasanton, CA, USA), PPAR-α (Lifespan Biosciences, Seattle, WA, USA), total-FoxO1, phospho-Ser^256^ FoxO1, total FoxO3a, phospho-Ser^253^ FoxO3a (Novus Biologicals, Centennial, CO, USA), nicotinamide adenine dinucleotide phosphate (NADPH) oxidase (Nox)1 and Nox4 (Lifespan Biosciences), Nox2 (BD Biosciences, San Jose, CA, USA), and HPRT1 (Thermo Fisher Scientific).

Equal aliquots of proteins extracted from GECs were electrophoresed on sodium dodecyl sulfate–polyacrylamide gels and electroblotted onto a nitrocellulose membrane (Millipore, Bedford, MA, USA). After blocking with ProNA Phospho-Block solution (TransLab, Daejeon, Republic of Korea) and hybridizing with primary antibodies, the membranes were incubated with a secondary antibody conjugated with horseradish peroxidase (Cell Signaling Technology). The immunoblot bands were visualized using a chemiluminescent detector (Amersham Pharmacia Biotech, London, UK) and the ChemiDoc™ XRS + system (Bio-Rad Laboratories). Glyceraldehyde 3-phosphate dehydrogenase (GAPDH) (Cell Signaling Technology) was used as an internal loading control.

### Measurement of oxidative stress and intracellular ROS

The lipid peroxidation of malondialdehyde (MDA) was assessed using the OxiSelect TBARS assay kit (Cell Biolabs Inc., San Diego, CA, USA) according to the manufacturer’s protocols. The OD of each protein sample at 540 nm was measured with a microplate reader (Bio-Rad Laboratories). Intracellular ROS production was measured by the nonfluorescent cell-permeating compound 2′-7′-dichlorofluorescein diacetate (DCF-DA) (Thermo Fisher Scientific). After 30 min of exposure to 10 μM DCF-DA, green fluorescence was visualized by fluorescence microscopy (Eclipse TE300; Nikon, Tokyo, Japan). The intensity of DCF-DA fluorescence was quantified using ImageJ software (U.S. National Institutes of Health, Bethesda, MD, USA).

### Measurement of purine metabolite concentrations

Hypoxanthine is metabolized into xanthine, which is metabolized into uric acid by XOR. These intracellular metabolites were quantified using a xanthine/hypoxanthine assay kit (Abcam, Cambridge, UK). Following the protocols, a reaction mixture of samples and standards was incubated for 30 min, and we measured the OD at 570 nm using a microplate reader (Bio-Rad Laboratories).

An AMP colorimetric assay kit and ATP colorimetric assay (BioVision Inc., Milpitas, CA, USA) was used to calculate the ratio of intracellular AMP to ATP, a key regulator of AMPK phosphorylation and purine metabolism, according to the manufacturer’s protocols. The OD of samples for intracellular AMP and ATP levels were measured at 570 nm with a microplate reader (Bio-Rad Laboratories). The intracellular AMP/ATP ratio was normalized to that of LG as a control.

### Enzyme immunoassay

XOR enzyme levels were double-checked using an XDH/XO enzyme-linked immunosorbent assay kit (Lifespan Biosciences). The enzyme levels of samples were measured at 450 nm and then compared to an OD standard curve generated using known antigen concentrations. The activity of superoxide dismutase (SOD) was assessed using an SOD activity assay kit (Abnova, Taipei City, Taiwan) in accordance with the manufacturer’s instructions. After incubation at 37 °C for 20 min, the color of the solution mixture with each sample and blank was measured at 450 nm using a microplate reader (Bio-Rad Laboratories). The SOD activity of the samples was determined using the equation provided in the manufacturer’s instructions.

### Statistical analysis

At least three independent experiments were performed for each analysis, and the results were presented as the mean ± standard deviation. Statistical significance between groups was assessed through analysis of variance, followed by the Tukey multiple comparison test (SPSS version 20.0, IBM Corp., Armonk, NY, USA). A level of *p* < 0.05 was considered to indicate statistically significance.

## Results

### XOR inhibition significantly increases cell survival in HG-treated GECs

**Figure 1:**
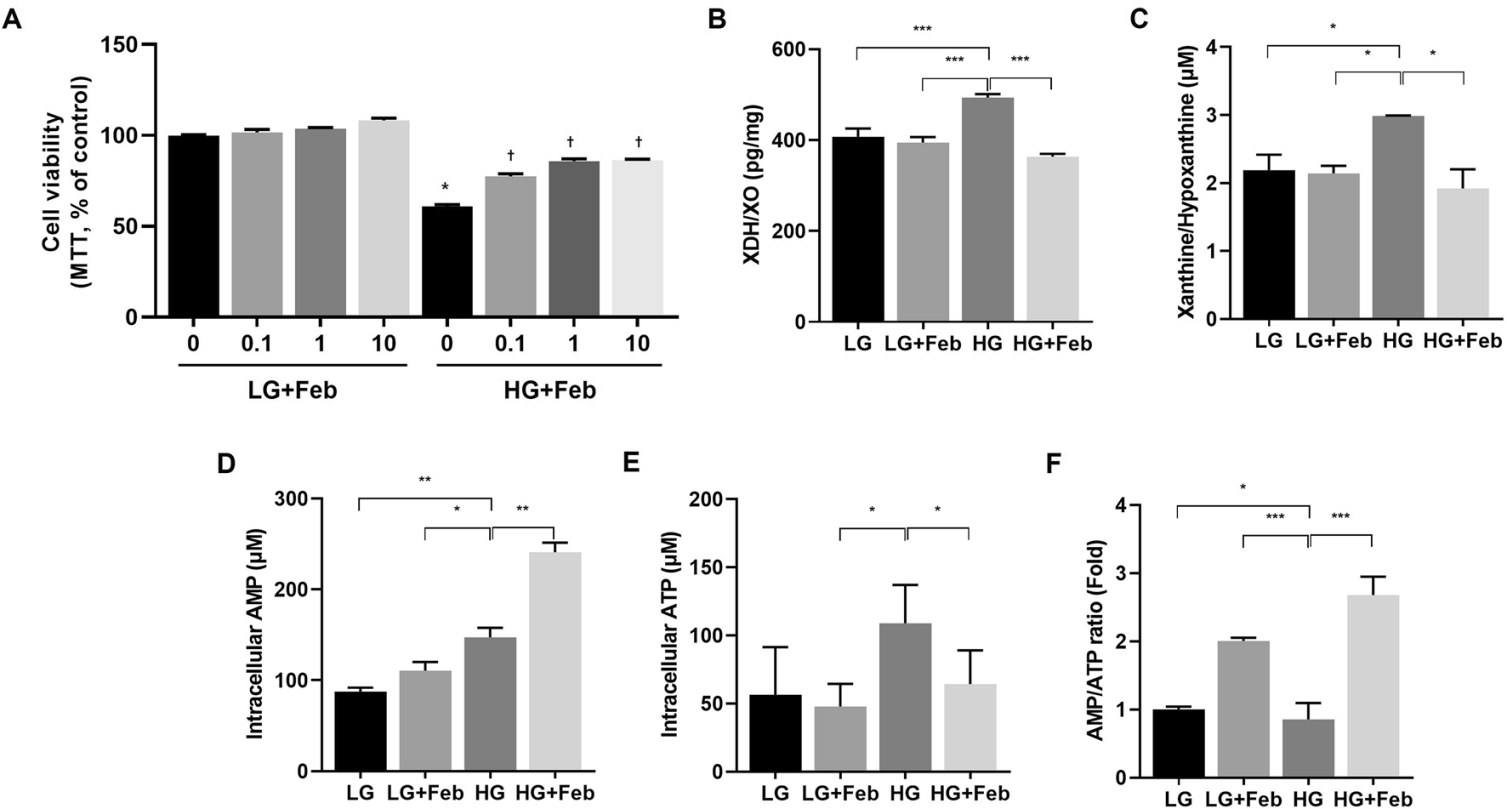
The effect of febuxostat treatment on cell viability, XDH/XO levels, xanthine/hypoxanthine levels, and AMP/ATP ratios in cultured human GECs treated with LG or HG medium. (**A**) Changes in cell viability measured by MTT assay in HG-treated GECs following dose-dependent febuxostat treatment. *p < 0.05 compared to LG medium, ^†^ *p* < 0.05 compared to HG medium. (**B**) XDH/XO levels, (**C**) xanthine/hypoxanthine levels, (**D**) intracellular AMP, (**E**) intracellular ATP, and (**F**) intracellular AMP/ATP ratio. *p < 0.05, **p < 0.01, ***p < 0.001 compared to other groups.

First, human GECs were treated with 0.1, 1, or 10 μM febuxostat for 48 h under both LG and HG conditions to decide the proper concentration for cytoprotection against the HG environment of febuxostat in GECs (Fig. 1A). Compared with those in the LG control group, the cell viability in the HG group substantially decreased by up to 60%. Febuxostat treatment did not alter cell survival under LG conditions, while cell survival increased after febuxostat treatment in a dose-dependent manner in HG environment. When the concentration of febuxostat exceeded 0.1 μM, the posttreatment cell viability significantly increased in GECs treated with HG medium (*p* < 0.01).

### XOR inhibition affects the purine salvage pathway and increases the AMP/ATP ratio in HG-treated GECs

The changes in XOR levels and the ratio of intracellular ATP to AMP induced by febuxostat treatment in HG-treated GECs were evaluated to investigate the mechanism by which XOR inhibition activates AMPK. XOR levels was not altered under LG conditions, but an increase in XOR levels was detected in the HG environment (*p* < 0.001, Fig. 1B). As expected, febuxostat significantly inhibited XOR levels in HG-treated GECs (*p* < 0.001, Fig. 1B). Xanthine/hypoxanthine levels were significantly increased in HG-treated GECs, and febuxostat treatment significantly suppressed xanthine/hypoxanthine levels in HG-treated GECs (*p* < 0.05, Fig. 1C).

XOR catalyzes the final two hydroxylation steps in purine degradation metabolism, activating the salvage pathway of purine base recycling to form purine nucleotides^17,28^. HG increased the intracellular AMP and ATP concentrations and decreased the intracellular AMP/ATP ratio (Fig. 1D–F), and febuxostat-mediated inhibition of XOR further increased the intracellular AMP concentration in HG-treated GECs (Fig. 1D). The ratio of intracellular AMP/ATP was increased by febuxostat treatment in both LG-treated and HG-treated GECs (*p* < 0.001, Fig. 1F).

### XOR inhibition increases AMPK phosphorylation and activates its downstream pathway in HG-treated GECs

**Figure 2:**
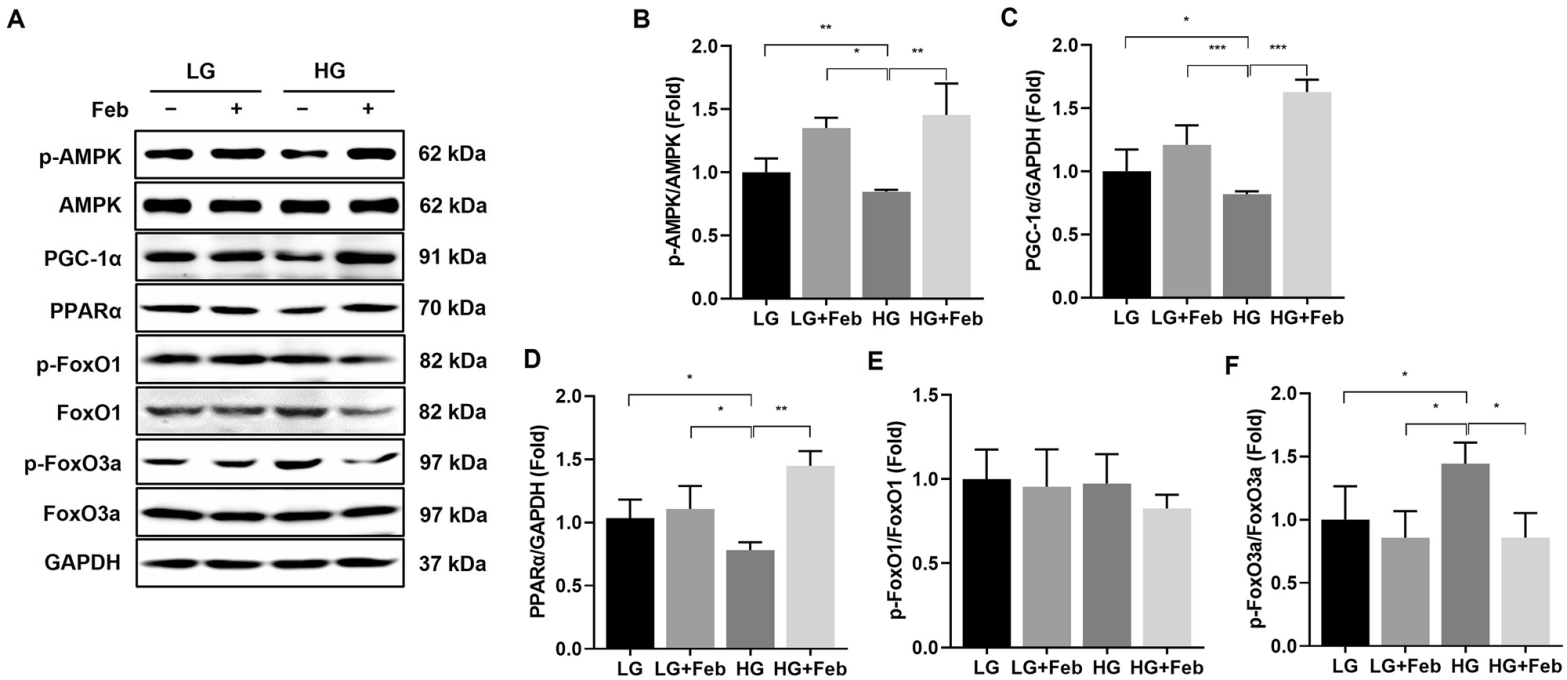
The effects of febuxostat treatment on AMPK, PGC-1α, PPAR-α, and FoxOs expressions in cultured human GECs treated with LG or HG medium. (**A**) Representative immunoblot images of AMPK, PGC-1α, PPAR-α, FoxO1, and FoxO3a. Results of quantitative analyses of the following are also shown: (**B**) phospho-Thr^172^ AMPK/total AMPK, (**C**) PGC-1α/GAPDH, (**D**) PPAR-α/GAPDH, (**E**) phospho-Ser^256^ FoxO1/total FoxO1, and (**F**) phospho-Ser^253^ FoxO3a/total FoxO3a. *p < 0.05, **p < 0.01, ***p < 0.001compared to other groups. Original blots are presented in Supplementary Fig. 1.

Next, the effects of XOR inhibition on HG-induced GEC injury via the activation of AMPK and its downstream pathway were evaluated in HG-treated GECs. While HG conditions significantly reduced the phospho-Thr^172^/total-AMPK ratio, febuxostat treatment effectively restored the phosphorylation of AMPK in HG-treated GECs (*p* < 0.01; Fig. 2A, B). HG conditions also suppressed PGC-1α and PPAR-α expression, but febuxostat treatment restored these expressions in HG-treated GECs (*p* < 0.001 and *p* < 0.01, respectively; Fig. 2A, C, D). Although there were no notable differences in the expression of phospho-Ser^256^/total FoxO1 among the experimental groups (Fig. 2A, E), the expression of phospho-Ser^253^/total FoxO3a increased under HG conditions and significantly decreased after febuxostat treatment (*p* < 0.05, Fig. 2A, F).

### XOR inhibition ameliorates oxidative stress by inhibiting NADPH oxidases and enhancing SOD in HG-treated GECs

**Figure 3:**
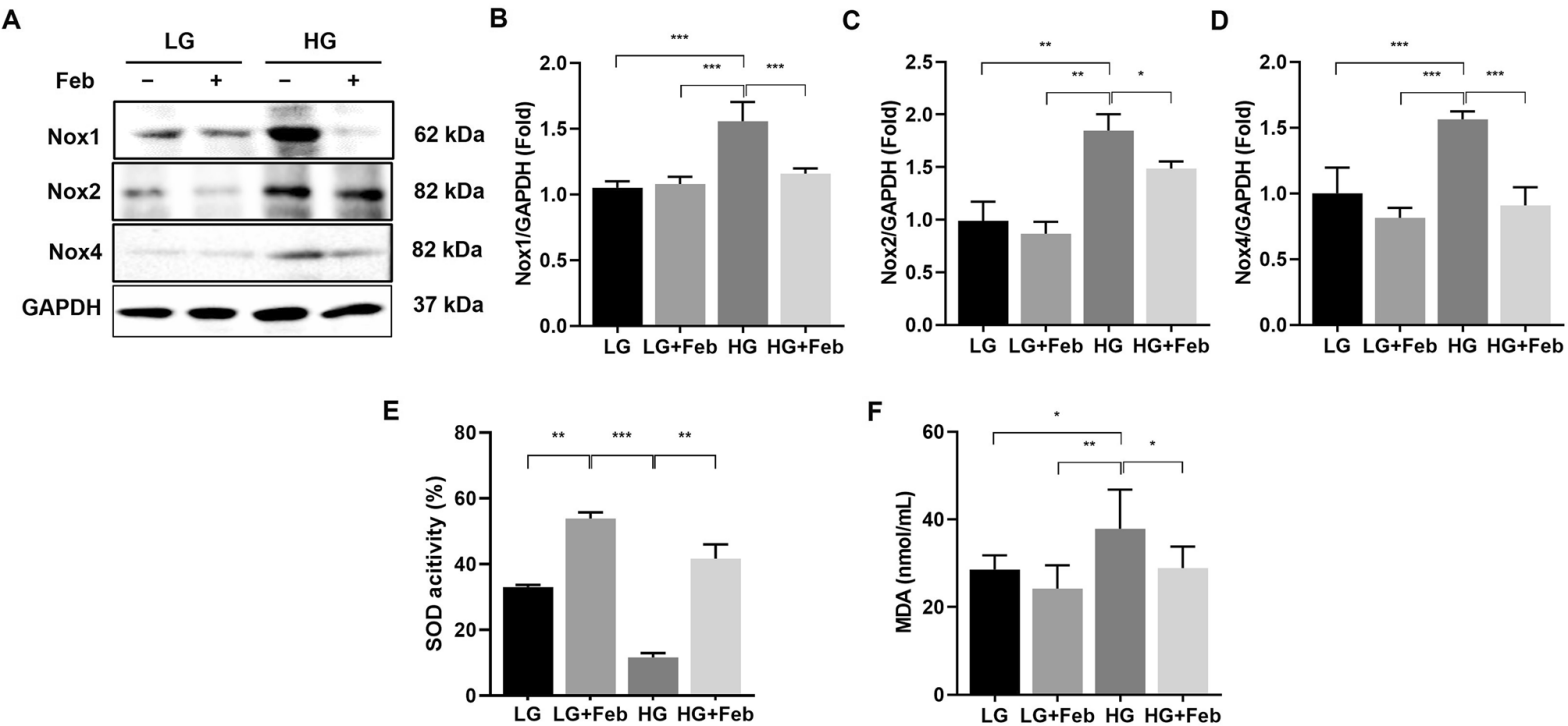
The effects of febuxostat treatment on Nox1, Nox2, and Nox4 expressions and changes in oxidative stress markers in cultured human GECs treated with LG or HG medium. (**A**) Representative immunoblot images of Nox1, Nox2, and Nox4. Results of quantitative analyses of the following are also shown: (**B**) Nox1/GAPDH, (**C**) Nox2/GAPDH, (**D**) Nox4/GAPDH, (**E**) SOD activities, and (**F**) MDA levels. *p < 0.05, **p < 0.01, ***p < 0.001 compared to other groups. Original blots are presented in Supplementary Fig. 2.

Oxidative stress in the HG environment is associated with increased NADPH oxidase and reduced activity of antioxidant enzymes in DKD^16^. The expressions of Nox1, Nox2, and Nox4 were substantially increased under HG conditions and significantly reduced by febuxostat treatment in HG-treated GECs (*p* < 0.001, *p* < 0.05, and *p* < 0.001, respectively; Fig. 3A–D). In addition, SOD activity was significantly lower in HG-treated GECs than in LG-treated GECs, and febuxostat treatment substantially enhanced SOD activity in HG-treated GECs (*p* < 0.01, Fig. 3E). The level of MDA, a useful biomarker for lipid peroxidation and oxidative stress, was markedly higher in HG-treated GECs than in LG-treated GECs, and febuxostat significantly reduced the MDA level (*p* < 0.05, Fig. 3F). These findings suggest that excess oxidative stress induced by the HG environment was alleviated by XOR inhibition.

### XOR inhibition ameliorates oxidative stress via AMPK-dependent signaling through the purine salvage pathway in HG-treated GECs

**Figure 4:**
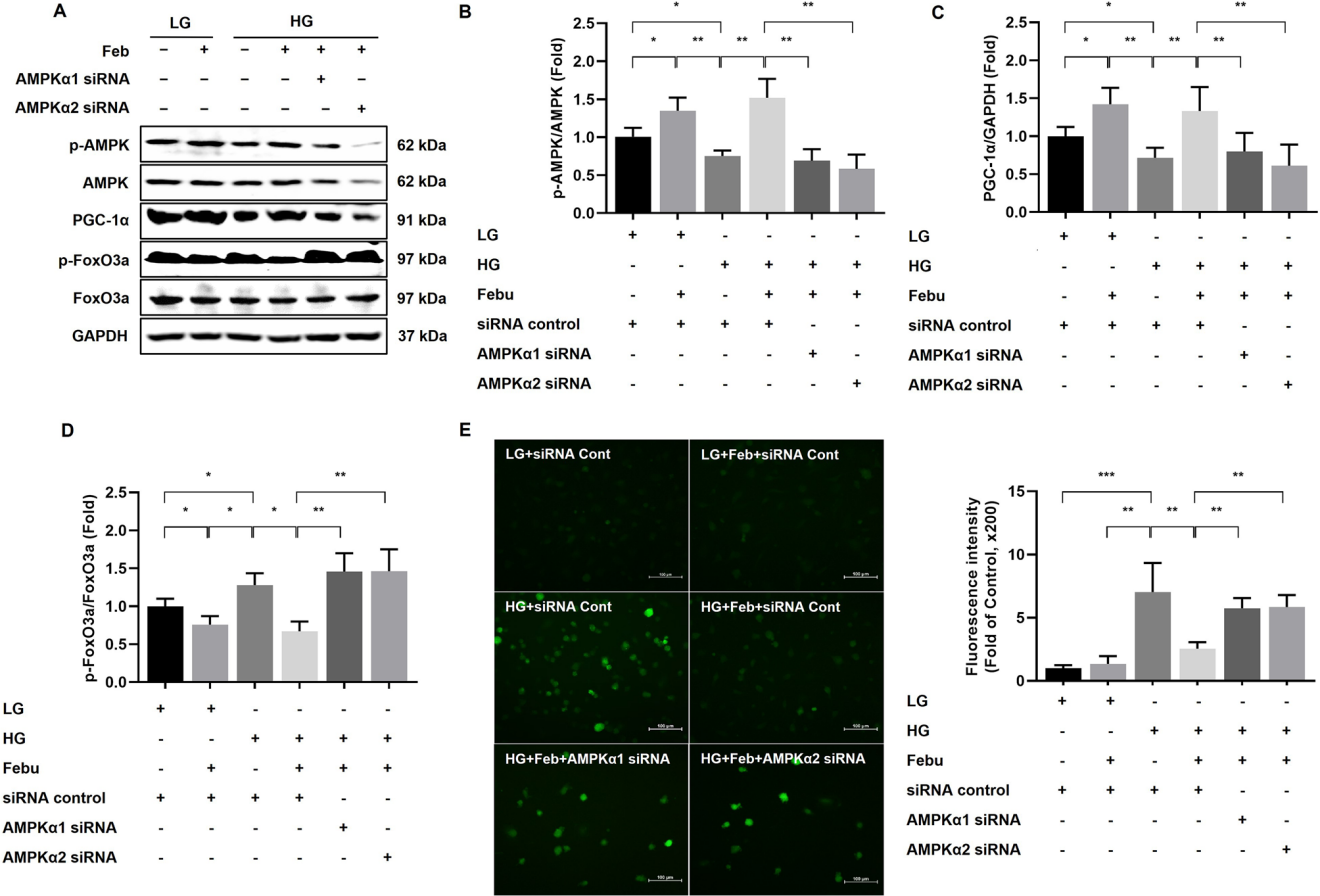
The effects of AMPKα1 or AMPKα2 siRNA knockdown on AMPK, PGC-1α, and FoxO3a expressions and ROS generation in HG-treated human GECs with or without febuxostat treatment. (**A**) Representative immunoblot images of AMPK, PGC-1α, and FoxO3a. Results of quantitative analyses of the following are also shown: (**B**) phospho-Thr^172^ AMPK/total AMPK, (**C**) PGC-1α/GAPDH, (**D**) phospho-Ser^253^ FoxO3a/total FoxO3a, and (**E**) intracellular ROS measured by DCF-DA assay. *p < 0.05, **p < 0.01, ***p < 0.001 compared to other groups. Original blots are presented in Supplementary Fig. 3.

To investigate whether XOR inhibition affects HG-induced GEC injury via AMPK-dependent signaling, further experiments on the knockdown of AMPK were conducted using siRNAs targeting AMPKα1 and AMPKα2 in LG- or HG-treated GECs. Despite the administration of febuxostat, the phosphorylation of AMPK in GECs treated with HG and transfected with siRNAs targeting AMPKα1 and AMPKα2 was substantially inhibited compared to that in HG-treated GECs transfected with control siRNA (*p* < 0.01, Fig. 4A, B). Consistent with the changes in phospho-Thr^172^/total-AMPK levels, the expression of PGC-1α was markedly suppressed in HG-treated GECs transfected with siRNAs targeting AMPKα1 and AMPKα2 compared to that in the siRNA control group despite febuxostat treatment (*p* < 0.01, Fig. 4A, C). In contrast, the phosphorylation of FoxO3a was further increased in GECs treated with HG and siRNAs targeting AMPKα1 and AMPKα2 compared to those in the siRNA control group despite febuxostat treatment (*p* < 0.05, Fig. 4A, D). HG exposure led to an increase in the number of DCF-DA–positive cells, and febuxostat treatment notably reduced the intracellular ROS levels in HG-treated GECs, as assessed by DCF-DA. The antioxidant effects of febuxostat treatment were eliminated by treatment with siRNAs targeting AMPKα1 and AMPKα2 in HG-treated GECs (*p* < 0.01; Fig. 4A, E).

**Figure 5:**
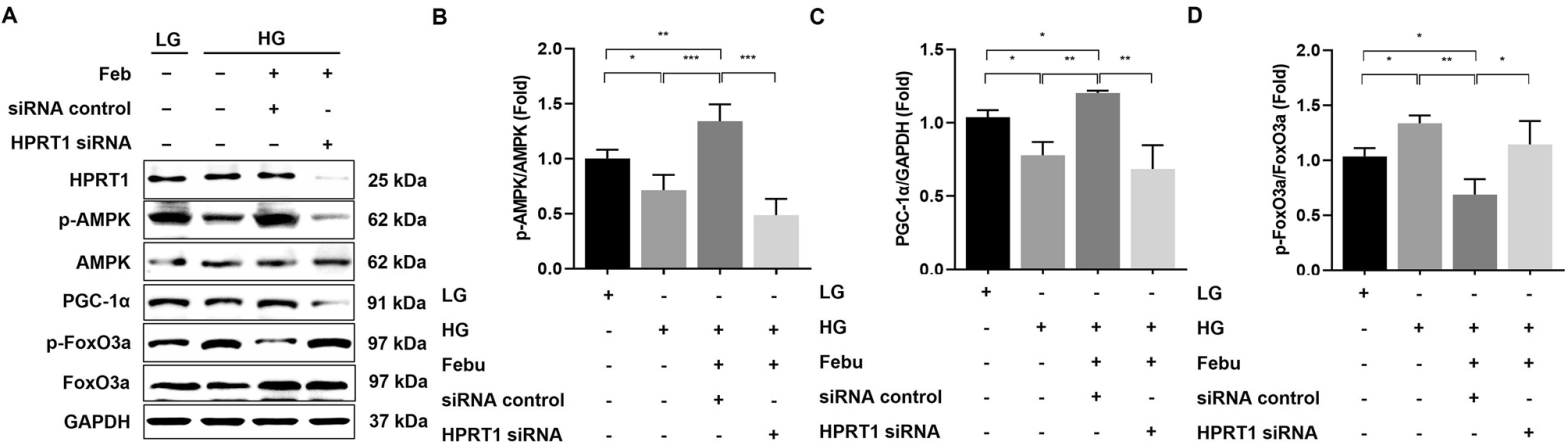
The effects of HPRT1 siRNA knockdown on AMPK, PGC-1α, and FoxO3a expressions in HG-treated human GECs with or without febuxostat treatment. (**A**) Representative immunoblot images of AMPK, PGC-1α, and FoxO3a. Results of quantitative analyses of the following are also shown: (**B**) phospho-Thr^172^ AMPK/total AMPK, (**C**) PGC-1α/GAPDH, and (**D**) phospho-Ser^253^ FoxO3a/total FoxO3a. *p < 0.05, **p < 0.01, ***p < 0.001 compared to other groups. Original blots are presented in Supplementary Fig. 4.

The changes in the expression of AMPK and its related downstream pathway induced by febuxostat treatment in HG-treated GECs transfected with siRNAs targeting HPRT1 were measured to confirm that the activation of AMPK by XOR inhibition is associated with the HPRT1-dependent purine salvage pathway. The phosphorylation of AMPK and PGC-1α was significantly lower in HG-treated GECs transfected with siRNAs targeting HPRT1 than in GECs in the siRNA control group despite febuxostat treatment (*p* < 0.001 and *p* < 0.001, Fig. 5A–C). On the other hand, the phosphorylation of FoxO3a was significantly higher in HG-treated GECs transfected with siRNAs targeting HPRT1 than that in the siRNA control group despite febuxostat treatment (*p* < 0.05; Fig. 5A, D).

## Discussion

This study demonstrated that XOR inhibition has a cytoprotective effect against HG-induced GEC injury by activating AMPK through the purine salvage pathway. Elevated XOR levels were observed in HG-induced GEC injury, correlated with a reduction in AMPK phosphorylation. These alterations resulted in the inhibition of PGC-1α and PPAR-α, subsequently leading to phosphorylation of FoxO3a and activation of Noxs, thereby increasing oxidative stress. Febuxostat treatment ameliorated HG-induced endothelial cell injury by activating AMPK phosphorylation and increasing the intracellular AMP/ATP ratio. These effects activate PGC-1α, dephosphorylate FoxO3a, and suppress Noxs, ultimately leading to the reversal of renal oxidative stress. These findings suggest that XOR inhibition improves oxidative stress against HG-induced GEC injury by activating the AMPK–PGC-1α–FoxO3a pathway through the purine salvage pathway.

Several previous studies have demonstrated that reducing the concentrations of purine metabolites, such as uric acid and XO, alleviates oxidative stress by activating AMPK^15,17–20^. Uric acid–induced oxidative stress stimulates insulin resistance, and AMPK activation by metformin treatment reduces uric acid–induced insulin resistance in skeletal muscle cells^18^. Kim et al.^19^ also revealed that febuxostat treatment suppressed endoplasmic reticulum stress by increasing AMPK activation in tunicamycin-treated proximal tubular cells and in a unilateral ureteral obstruction mouse model. Our previous study demonstrated that contrast-induced oxidative stress significantly deteriorated kidney function and increased XOR, while febuxostat ameliorated AKI by up-regulating AMPK phosphorylation^15^. In the present study, we investigated the effects of XOR inhibition on the AMPK-dependent signaling pathway in HG-GEC injury. The protective effects of febuxostat on HG-induced intracellular ROS were attenuated by AMPK-α1/2 siRNA. To the best of our knowledge, this study is the first to confirm the beneficial role of XOR inhibition through AMPK activation in GEC injury associated with HG-induced oxidative stress.

AMPK is the master regulator of cellular energy homeostasis, restoring energy balance during metabolic stress by balancing the intracellular AMP/ATP ratio^21^. Under energy-depleted conditions, the intracellular AMP level increases, while the ATP level decreases, leading to an increase in the AMP/ATP ratio and activation of AMPK to restore the cellular ATP level through downregulation of anabolic pathways and upregulation of catabolic pathways^22^. In energy-excess states, such as HG, AMPK activation is reduced to stimulate protein synthesis, cell growth, and storage^22^. Interestingly, recent studies have shown that XOR inhibition prevents AKI by restoring intracellular ATP levels in model of renal ischemia–reperfusion injury^23,24^. Some experiments have also shown that febuxostat significantly increases intracellular AMP levels in models of niacin-induced cell injury or oxonic acid–induced renal tubular injury^25,26^. In the present study, the intracellular AMP/ATP ratio decreased under HG conditions, and febuxostat-induced XOR inhibition mainly stimulated the intracellular AMP levels and ultimately activated AMPK by increasing the intracellular AMP/ATP ratio. However, our results do not suggest a direct recovery of intracellular ATP levels by XOR inhibition in HG-treated GECs. These discrepancies can be explained by interstudy differences in experimental design, including exposure time to stimuli and treatment duration, as well as in disease models such as DKD or ischemia–reperfusion injury.

Purine nucleotides are involved in various cellular functions, including energy storage and transportation, participation in signaling pathways, and serving as both nucleic acid precursors and cofactors in numerous metabolic reactions^27^. In purine catabolism, XOR plays an important role in generating irreversible byproducts, such as xanthine and uric acid, that block the purine salvage pathway^28^. Therefore, XOR inhibition can reduce the production of XOR-derived ROS by promoting the reuse of hypoxanthine and ATP production through the purine salvage pathway^28^. HPRT plays a vital role as an enzyme in the purine salvage pathway, and Lesch–Nyhan disease, an inherited metabolic disorder disrupting the recycling of ATP, is caused by mutations in the HPRT1 gene^29^. A recent study demonstrated that XOR inhibition by febuxostat facilitates the conversion from hypoxanthine to inosine monophosphate by HPRT, and the beneficial effect of febuxostat was canceled by silencing the HPRT1 gene in cultured renal tubular cells^23^. The present study also revealed that the activation of AMPK–PGC-1α–FoxO3a signaling by febuxostat treatment was abolished by the knockdown of HPRT1 using HPRT1 siRNA. This finding suggested that XOR inhibition increases the activation of AMPK through the HPRT1-dependent purine salvage pathway.

In the present study, we focused on the relationships among AMPK, PGC-1α, Noxs (Nox1, Nox2, and Nox4), and FoxOs as components of the cellular response to oxidative stress in DKD. PGC-1α acts as the master transcriptional regulator, participating in mitochondrial biogenesis, oxidative phosphorylation, carbohydrate and lipid metabolism, and the detoxification of ROS^30^. AMPK can directly interact with and activate PGC-1α, and the activation of AMPK promotes PGC-1α–dependent antioxidant responses^31,32^. PGC-1α also regulates FoxOs activity in various systems, especially gluconeogenesis and cellular antioxidant defense^30,33^. Among the FoxO family members, FoxO3a is a direct target of PGC-1α and physically interacts with PGC-1α to regulate antioxidant gene expression^34^. In addition, AMPK can directly regulate FoxO3a, and the AMPK–FoxO3a signaling pathway activates antioxidant enzymes, including thioredoxin, peroxiredoxin, SOD2, and catalase, which can reduce ROS production^35^. AMPK also plays a crucial role in regulating the Nox system, and several experimental studies have highlighted that AMPK activation also reduces oxidative stress by suppressing Nox-derived ROS—in particular, Nox4-derived ROS—to protect against kidney injury^36–38^. Our study confirmed that the AMPK activation induced by XOR inhibition significantly upregulated PGC-1α and FoxO3a and suppressed Nox1, Nox2, and Nox4. Furthermore, the knockdown of AMPK offset the activation of PGC-1α and FoxO3a and the attenuation of ROS production in HG-induced GECs. These findings indicate that the protective mechanism of XOR inhibition is facilitated by activating AMPK and its downstream pathway, which are linked to the antioxidant defense mechanism in HG-induced glomerular endothelial injury.

**Figure 6:**
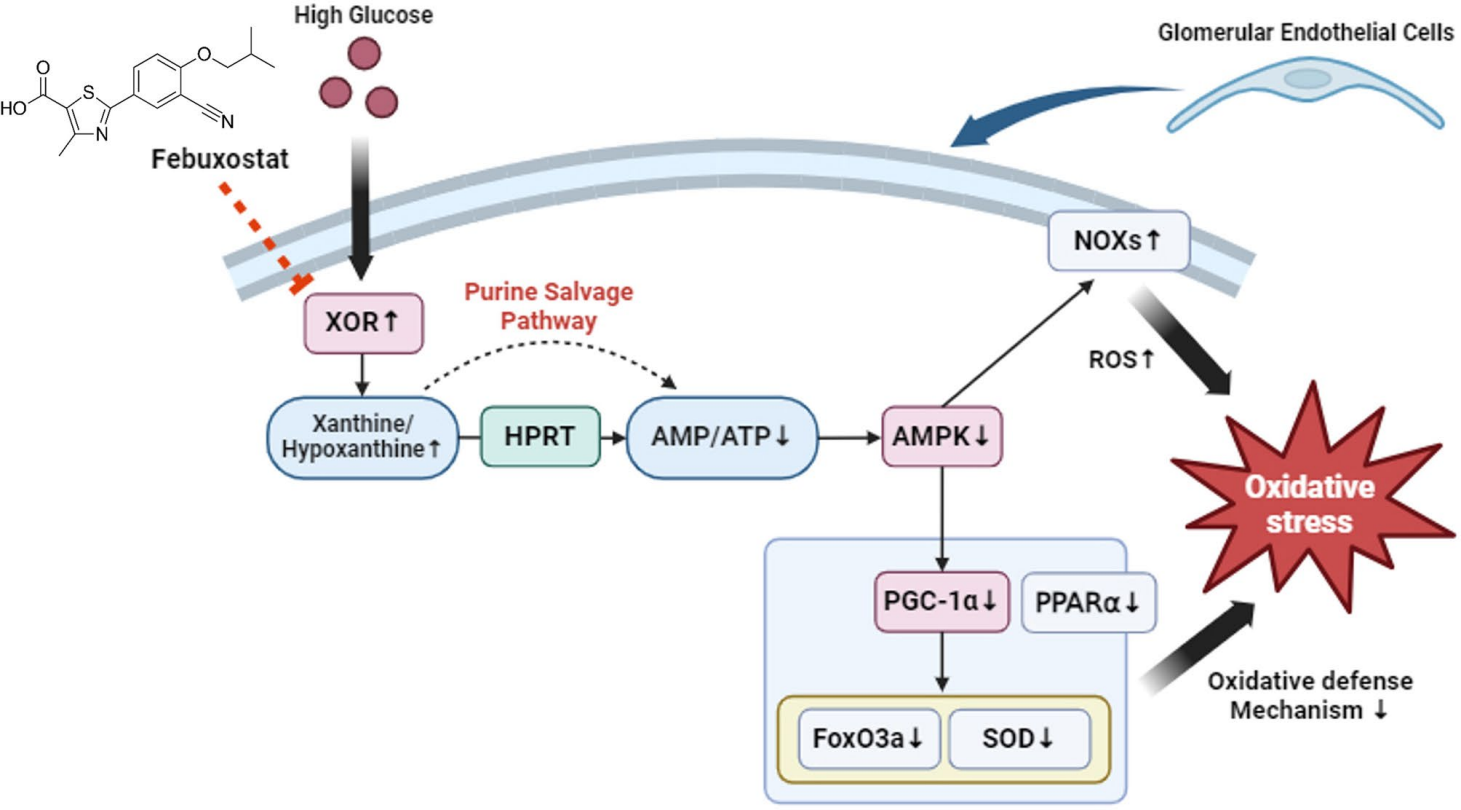
Schematic illustration of the proposed molecular mechanism for cytoprotective effects of XOR inhibition by febuxostat treatment through the purine salvage pathway in HG-induced GEC injury. Created with BioRender.com.

Although this study clearly demonstrated the cytoprotective effect of XOR inhibition and the underlying mechanism of AMPK activation using genetically knockdown cells, the lack of evidence using in vivo experiments is a limitation of the study. To finally confirm the therapeutic role of XOR inhibition in DKD, further well-designed in vivo experiments and clinical trials may be needed to explore whether febuxostat acts on the renoprotective effects of reducing ROS through these signaling pathways. Figure 6 shows a schematic representation of the suggested molecular mechanism underlying the cytoprotective effects of XOR inhibition on HG-induced endothelial cell injury.

## Conclusions

XOR inhibition protects against HG-induced GEC injury through the upregulation of AMPK–PGC-1α–FoxO3a signaling via the HPRT1-dependent purine salvage pathway. These findings highlight that the inhibition of XOR via AMPK activation protects GECs from HG-derived ROS and may be a therapeutic target for preventing the progression of DKD.

### Supplementary Information

Supplementary Figures.

## Data Availability

The datasets generated and analyzed in the current study are available from the corresponding author upon reasonable request.

## References

[1] Hong YA (2021). Trends in epidemiologic characteristics of end-stage renal disease from 2019 Korean renal data system (KORDS). Kidney Res. Clin. Pract..

[2] Tuttle KR (2022). Molecular mechanisms and therapeutic targets for diabetic kidney disease. Kidney Int..

[3] Jha JC, Ho F, Dan C, Jandeleit-Dahm K (2018). A causal link between oxidative stress and inflammation in cardiovascular and renal complications of diabetes. Clin. Sci. (Lond.).

[4] Lassén E, Daehn IS (2020). Molecular mechanisms in early diabetic kidney disease: Glomerular endothelial cell dysfunction. Int. J. Mol. Sci..

[5] Maiuolo J, Oppedisano F, Gratteri S, Muscoli C, Mollace V (2016). Regulation of uric acid metabolism and excretion. Int. J. Cardiol..

[6] Nishino T, Okamoto K (2015). Mechanistic insights into xanthine oxidoreductase from development studies of candidate drugs to treat hyperuricemia and gout. J. Biol. Inorg. Chem..

[7] Eleftheriadis T, Pissas G, Antoniadi G, Liakopoulos V, Stefanidis I (2018). Allopurinol protects human glomerular endothelial cells from high glucose-induced reactive oxygen species generation, p53 overexpression and endothelial dysfunction. Int. Urol. Nephrol..

[8] Itano S (2020). Non-purine selective xanthine oxidase inhibitor ameliorates glomerular endothelial injury in Ins(Akita) diabetic mice. Am. J. Physiol. Ren. Physiol..

[9] Liu J, Wang C, Liu F, Lu Y, Cheng J (2015). Metabonomics revealed xanthine oxidase-induced oxidative stress and inflammation in the pathogenesis of diabetic nephropathy. Anal. Bioanal. Chem..

[10] Hasan M (2022). Assessment of the relationship between serum xanthine oxidase levels and type 2 diabetes: A cross-sectional study. Sci. Rep..

[11] Nakatani S (2021). Plasma xanthine oxidoreductase activity associated with glycemic control in patients with pre-dialysis chronic kidney disease. Kidney Blood Press. Res..

[12] Washio K (2020). Xanthine oxidoreductase activity correlates with vascular endothelial dysfunction in patients with type 1 diabetes. Acta Diabetol..

[13] Hallows KR, Mount PF, Pastor-Soler NM, Power DA (2010). Role of the energy sensor AMP-activated protein kinase in renal physiology and disease. Am. J. Physiol. Ren. Physiol..

[14] Hardie DG, Scott JW, Pan DA, Hudson ER (2003). Management of cellular energy by the AMP-activated protein kinase system. FEBS Lett..

[15] Yang KJ (2019). Inhibition of xanthine oxidoreductase protects against contrast-induced renal tubular injury by activating adenosine monophosphate-activated protein kinase. Free Radic. Biol. Med..

[16] Jha JC, Banal C, Chow BS, Cooper ME, Jandeleit-Dahm K (2016). Diabetes and kidney disease: Role of oxidative stress. Antioxid. Redox Signal..

[17] Zhang Y (2013). Uric acid induces oxidative stress and growth inhibition by activating adenosine monophosphate-activated protein kinase and extracellular signal-regulated kinase signal pathways in pancreatic beta cells. Mol. Cell. Endocrinol..

[18] Yuan H (2017). Metformin ameliorates high uric acid-induced insulin resistance in skeletal muscle cells. Mol. Cell. Endocrinol..

[19] Kim H, Baek CH, Chang JW, Yang WS, Lee SK (2020). Febuxostat, a novel inhibitor of xanthine oxidase, reduces ER stress through upregulation of SIRT1-AMPK-HO-1/thioredoxin expression. Clin. Exp. Nephrol..

[20] Cicerchi C (2014). Uric acid-dependent inhibition of AMP kinase induces hepatic glucose production in diabetes and starvation: Evolutionary implications of the uricase loss in hominids. FASEB J..

[21] Garcia D, Shaw RJ (2017). AMPK: Mechanisms of cellular energy sensing and restoration of metabolic balance. Mol. Cell..

[22] Kume S, Thomas MC, Koya D (2012). Nutrient sensing, autophagy, and diabetic nephropathy. Diabetes.

[23] Fujii K (2019). Xanthine oxidase inhibitor ameliorates postischemic renal injury in mice by promoting resynthesis of adenine nucleotides. JCI Insight.

[24] Tani T, Okamoto K, Fujiwara M, Katayama A, Tsuruoka S (2019). Metabolomics analysis elucidates unique influences on purine/pyrimidine metabolism by xanthine oxidoreductase inhibitors in a rat model of renal ischemia-reperfusion injury. Mol. Med..

[25] Nomura J, Kobayashi T, So A, Busso N (2019). Febuxostat, a xanthine oxidoreductase inhibitor, decreases NLRP3-dependent Inflammation in macrophages by activating the purine salvage pathway and restoring cellular bioenergetics. Sci. Rep..

[26] Xiao J (2019). AMPK alleviates high uric acid-induced Na(+)-K(+)-ATPase signaling impairment and cell injury in renal tubules. Exp. Mol Med..

[27] Camici M, Allegrini S, Tozzi MG (2018). Interplay between adenylate metabolizing enzymes and AMP-activated protein kinase. FEBS J..

[28] Furuhashi M (2020). New insights into purine metabolism in metabolic diseases: Role of xanthine oxidoreductase activity. Am. J. Physiol. Endocrinol. Metab..

[29] Lesch M, Nyhan WL (1964). A Familial disorder of uric acid metabolism and central nervous system function. Am. J. Med..

[30] Liang H, Ward WF (2006). PGC-1alpha: A key regulator of energy metabolism. Adv. Physiol. Educ..

[31] Cantó C, Auwerx J (2009). PGC-1alpha, SIRT1 and AMPK, an energy sensing network that controls energy expenditure. Curr. Opin. Lipidol..

[32] Rabinovitch RC (2017). AMPK maintains cellular metabolic homeostasis through regulation of mitochondrial reactive oxygen species. Cell Rep..

[33] Klotz LO (2015). Redox regulation of FoxO transcription factors. Redox Biol..

[34] Rius-Pérez S, Torres-Cuevas I, Millán I, Ortega ÁL, Pérez S (2020). PGC-1α, inflammation, and oxidative stress: An integrative view in metabolism. Oxid. Med. Cell Longev..

[35] Wu SB, Wu YT, Wu TP, Wei YH (1840). Role of AMPK-mediated adaptive responses in human cells with mitochondrial dysfunction to oxidative stress. Biochim. Biophys. Acta.

[36] Sharma K (2014). Obesity, oxidative stress, and fibrosis in chronic kidney disease. Kidney Int. Suppl..

[37] Lee HJ (2017). Hydrogen sulfide inhibits high glucose-induced NADPH oxidase 4 expression and matrix increase by recruiting inducible nitric oxide synthase in kidney proximal tubular epithelial cells. J. Biol. Chem..

[38] Lu XH, Zhang J, Xiong Q (2022). Suppressive effect erythropoietin on oxidative stress by targeting AMPK/Nox4/ROS pathway in renal ischemia reperfusion injury. Transpl. Immunol..

