# Supplementary material for: Xanthine oxidoreductase inhibition ameliorates high glucose-induced glomerular endothelial injury by activating AMPK through the purine salvage pathway
Source: Sci Rep. 2024 May 15;14:11167. doi: 10.1038/s41598-024-61436-1 (PMC11096301; doi:10.1038/s41598-024-61436-1)

Supplementary Figure 1

A

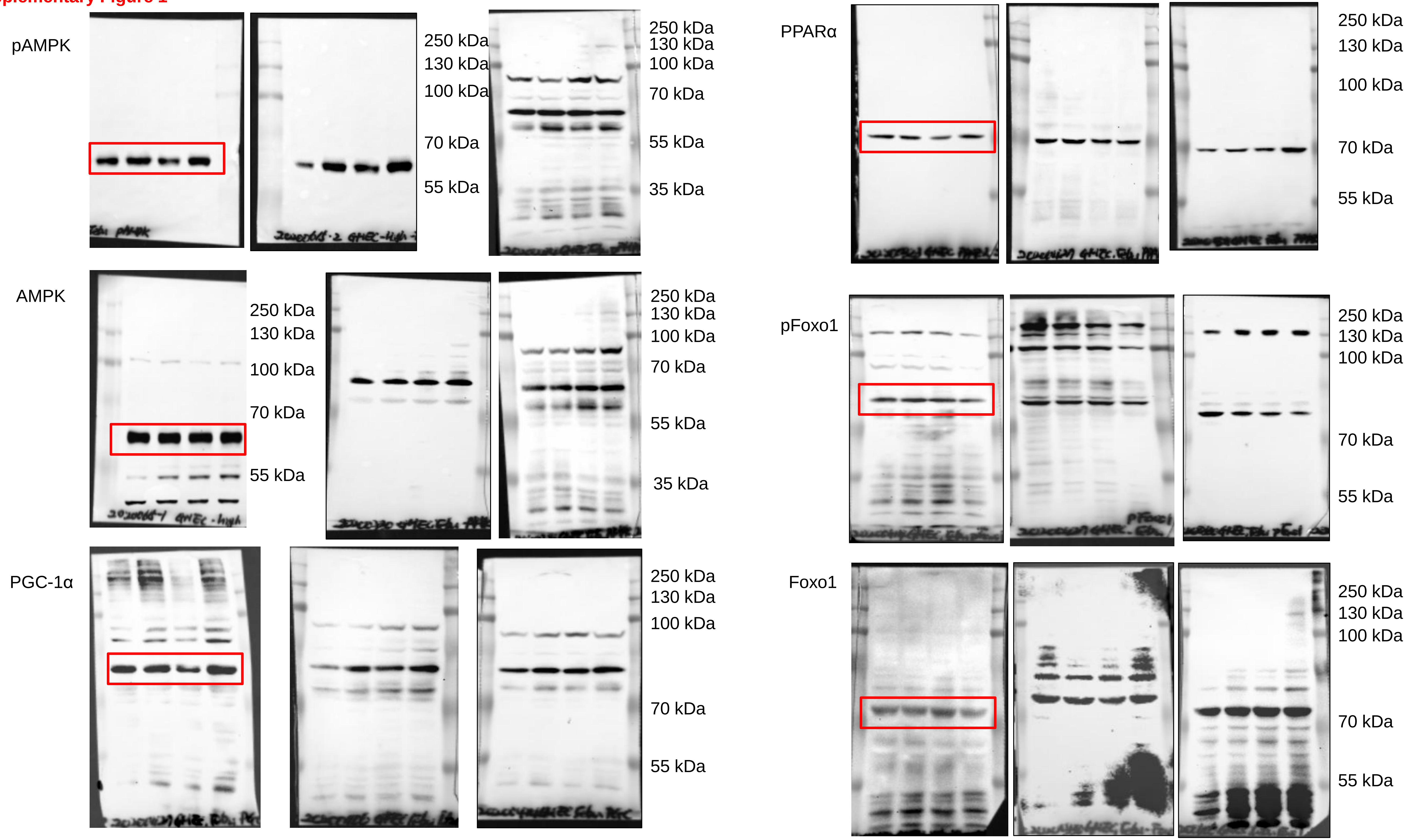

Supplementary Figure 1

A

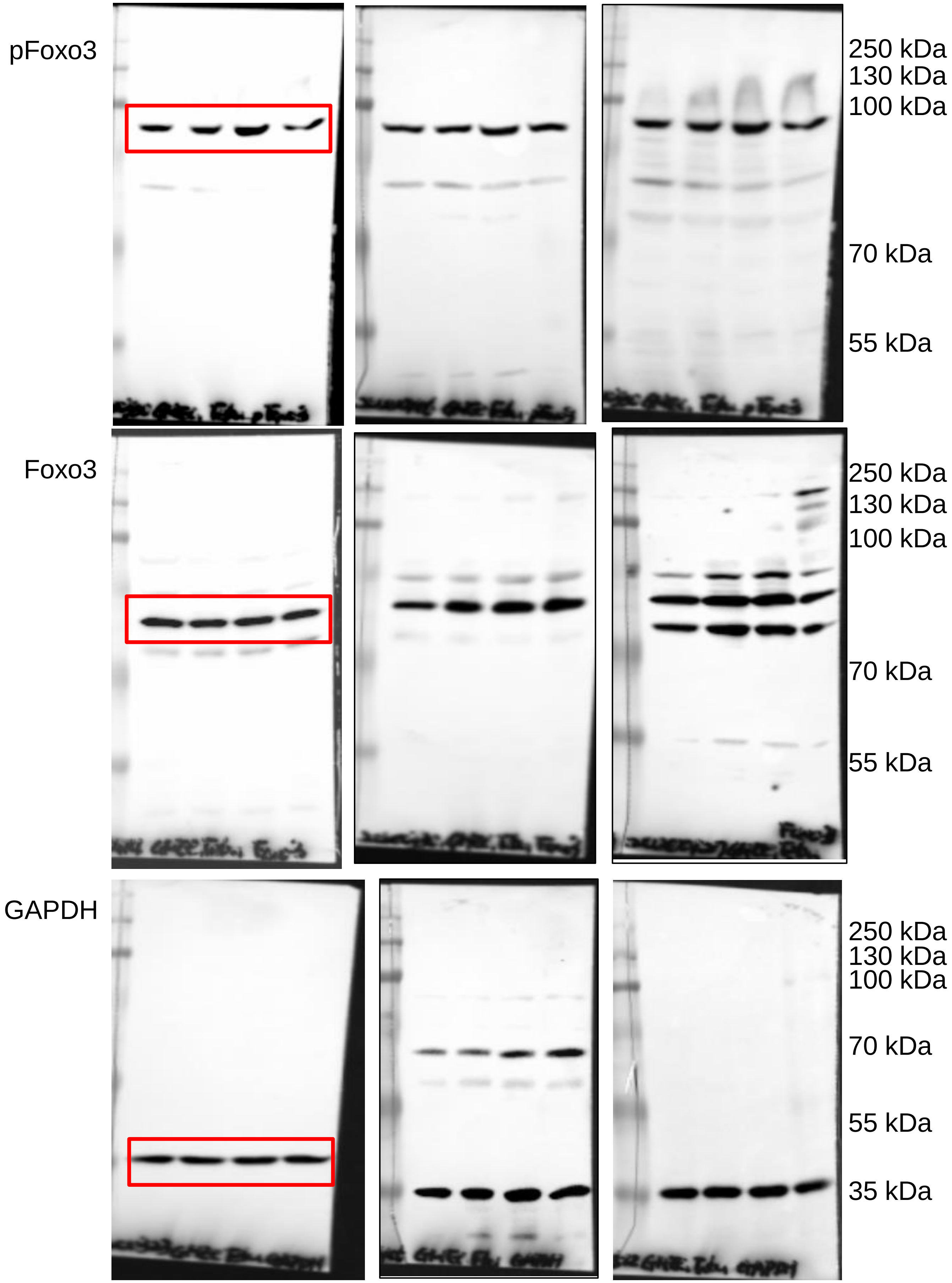

Supplementary Figure 2

A

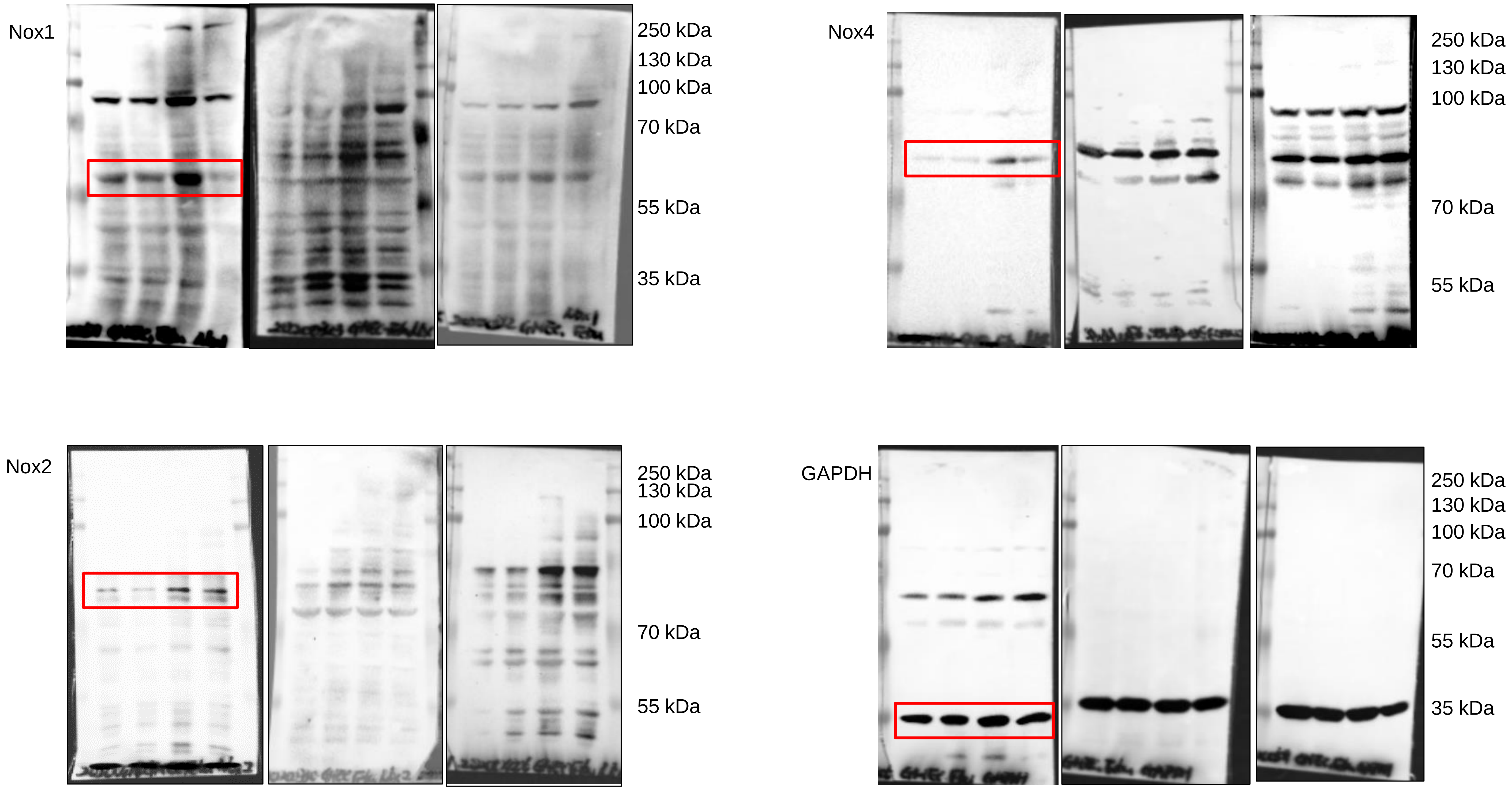

Supplementary Figure 3

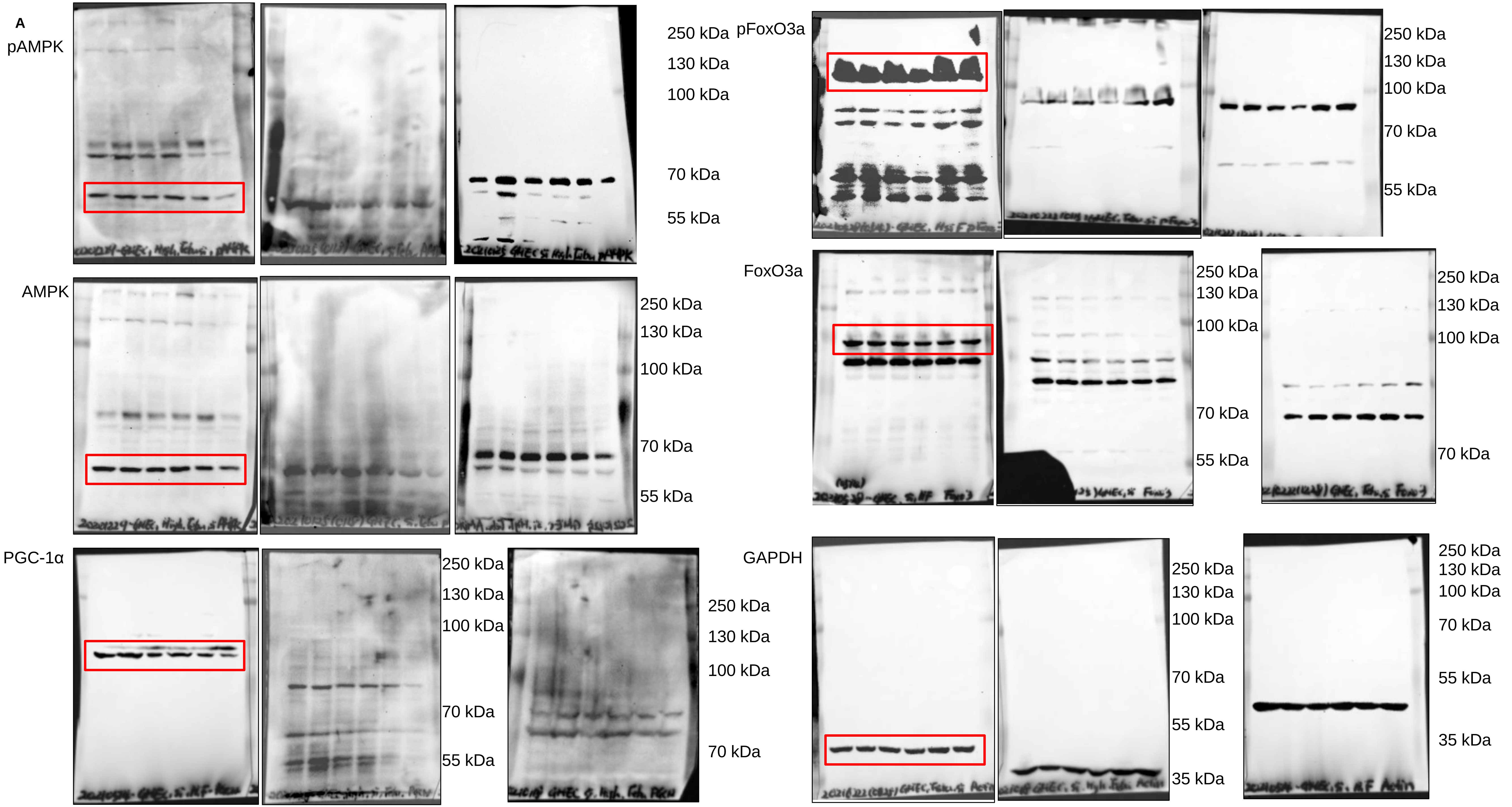

Supplementary Figure 4

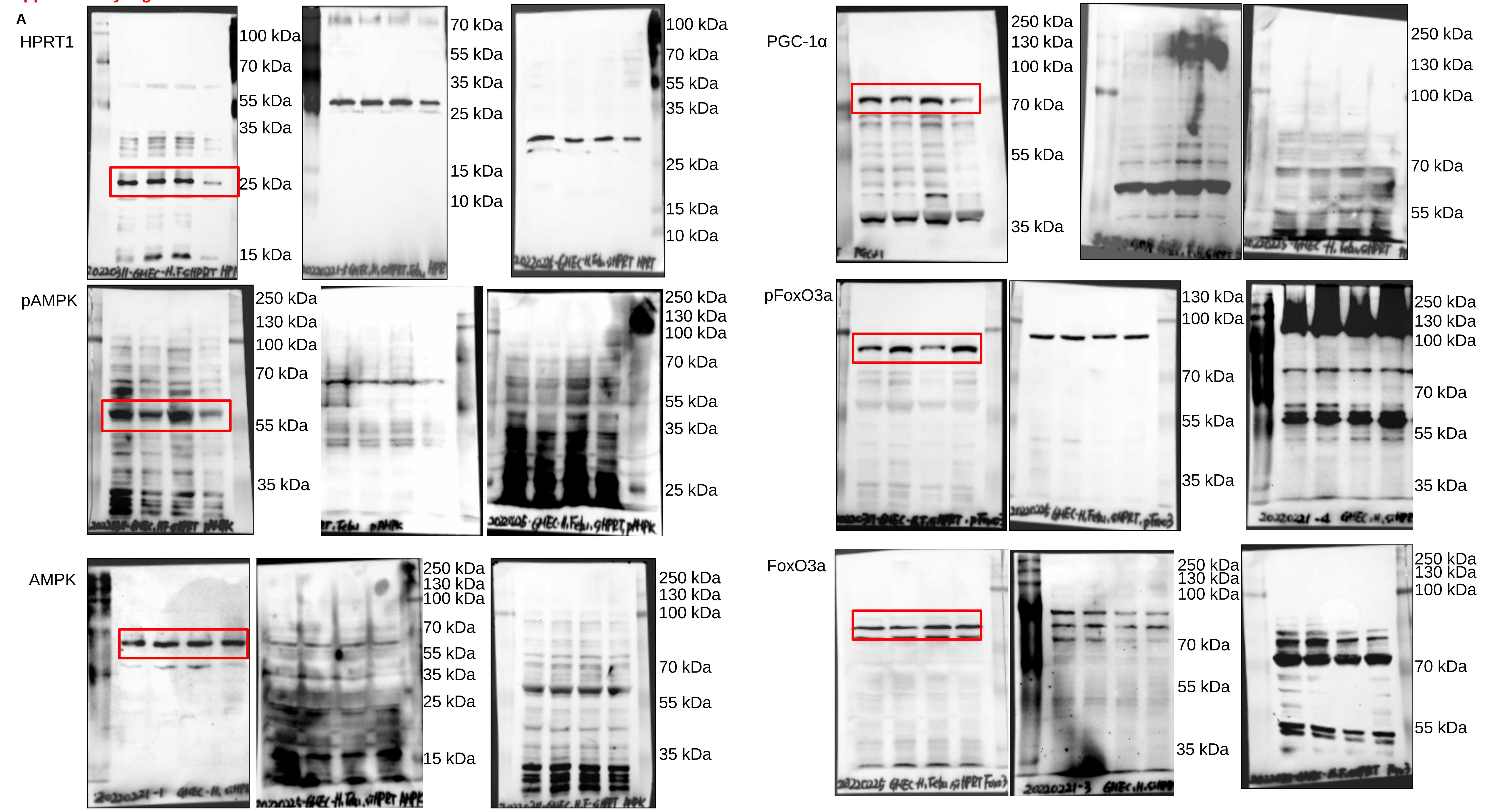

Supplementary Figure 4

A

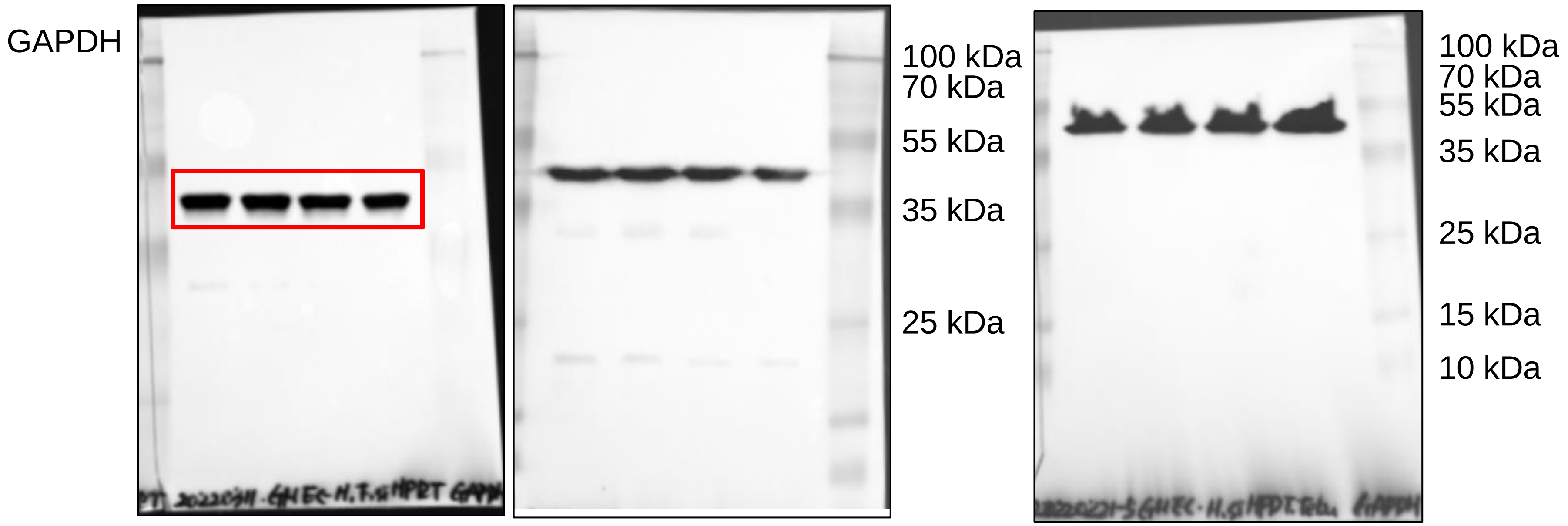

Supplement: Supplementary file 1 — Supplementary Figures. [file 41598_2024_61436_MOESM1_ESM.pdf]
